# Pathophysiology of the Serotonin System in the Nervous System and Beyond

**DOI:** 10.3390/ijms23094712

**Published:** 2022-04-25

**Authors:** Philippe De Deurwaerdère, Giuseppe Di Giovanni

**Affiliations:** 1 Centre National de la Recherche Scientifique, UMR CNRS 5287, 146 rue Léo Saignat, B.P.281, CEDEX, F-33000 Bordeaux, France; 2 Laboratory of Neurophysiology, Department of Physiology and Biochemistry, Faculty of Medicine and Surgery, University of Malta, MSD2080 Msida, Malta; 3 Neuroscience Division, School of Biosciences, Cardiff University, Cardiff CF10 3AX, UK

Serotonin (5-HT) is an attractive neurotransmitter system, in terms of physiology, physiopathology, and medicines. Here, we furthered our first Special Issue, published in IJMS “Serotonin in health and diseases”, with this entitled “Pathophysiology of the Serotonin System in the Nervous System and Beyond”. This collection of articles dedicated to serotonin encompasses 13 contributions, made up of 8 original research articles, and 5 reviews. It covers several aspects of the pathophysiology of the serotonin system at the periphery (lung disease or irritable bowel syndrome) and the CNS (anxiety, depression, addiction, epilepsy, neurotoxicity, memory and cognitive deficits). The influence of several serotonergic receptors is highlighted in this collection of articles, with some emphasis on 5-HT_1A_, 5-HT_2A/C_, 5-HT_4_ or 5-HT_6_ receptor subtypes. Different levels of understanding of the serotonergic transmission are proposed herein, from the molecular partners of serotonergic receptors modifying the activity of the receptor to its constitutive activity, to the cellular organization in the brain.

We believe that 5-HT is a special neurochemical system in living organisms, involved with different levels of biological complexity, from intimate molecular aspects to higher brain function. Hitherto, it is still a mysterious system, despite the numerous data released per year. The Special Issue presented herein, “Pathophysiology of the Serotonin System in the Nervous System and Beyond”, follows the “Serotonin in health and diseases” published two years ago in IJMS [1]. It covers several aspects of the pathophysiology of the 5-HT system at the periphery (lung disease or irritable bowel syndrome) and the central nervous system (hyperthermia, anxiety, depression, addiction, epilepsy, neurotoxicity, memory and cognitive deficits).

The 5-HT receptors undergo various regulatory processes and these modulatory actions confer distinct properties to the receptor. The field of 5-HT_1A_ receptors is particularly interesting because it could end up in the amelioration of anxiolytic, antidepressant, and antipsychotic drugs. Gupta et al. notably reported that the SUMOylation of the 5-HT_1A_ receptor is a dynamic regulation, possibly involving two opposite regulators, namely PIASxα and SENP2 [2]. These two proteins are distinctly recruited upon the administration of a 5-HT_1A_ receptor agonist and/or 17β-estradiol-3-benzoate. It reminds us that the 5-HT_1A_ receptor is subject to another type of regulation: dimerization. Borroto-Escuela et al. wrote a review article on the heterodimers and their role in the pathophysiology of depression. A large part is dedicated to 5-HT_1A_ auto- and hetero-receptors and their physical interaction with FGF receptors or 5-HT_2A_ receptors [3]. They also highlighted some heterocomplexes, involving oxytocin receptors and 5-HT_2A_ and 5-HT_2C_ receptors. Dysregulation of these heterodimers could participate in the development of major depressive disorders.

The 5-HT_2A_ and 5-HT_2C_ receptors are the targets of serotonergic-based psychedelic drugs, which are proposed to be antidepressants. Raval et al. focused on the 5-HT_2A_ receptor and plasticity induced by a single, low dose of psilocybin in pigs [4]. Using quantitative autoradiography in a well-designed study, the authors provided evidence that psilocybin reduced the density of 5-HT_2A_ receptors in the hippocampus and prefrontal cortex, at 1 but not 7 days after injection. Conversely, they reported an increased density of SV2A, used as a marker of synaptic plasticity, at 7 but not one days after psilocybin injection. This synaptic reorganization could participate in the mid/long-term antidepressant effects of psychedelics. Other factors interacting with 5-HT could be involved in antidepressant effects, such as the brain-derived neurotrophic factor (BDNF). Diniz et al. studied the effects resulting from an overexpression of BDNF in the ventral hippocampus in SERT−/− rats, a rat model characterized by low levels of BDNF and depressive-like phenotypes [5]. They provided evidence that the upregulation of BDNF opposed depressive-like phenotypes in the sucrose preference test or the forced swim test, increased social interaction, and blunted corticosterone response in a restraint procedure.

Numerous 5-HT receptors participate in cognitive processes and alteration of their activities might sustain cognitive deficits. Doucet et al. reported strong evidence for the aberrant constitutive activity of 5-HT_6_ receptors in a mouse model of neurofibromatosis type 1 (NF1), the nf1+/− mice [6]. This model resumes some of the cognitive deficits of the human disease. They found that the mTOR pathway was hyperactive in this model. More importantly, the 5-HT_6_ receptor inverse agonist SB258585 or rapamycin itself, but not the 5-HT_6_ receptor neutral antagonist CPPQ, suppressed cognitive deficits. The data suggest that the improper expression of the nf gene promotes the constitutive activity of 5-HT_6_ receptors towards the mTOR signaling pathway. The appropriate therapeutic response lies in the use of inverse agonists, acting at the 5-HT_6_ receptor. In addition to the 5-HT_6_ receptor as a target in cognitive deficits, several studies have proposed to focus on 5-HT_4_ receptors. As recalled by Roux et al. [7], the 5-HT_4_ receptors are expressed in the hippocampus and evidence has been provided for the pro-cognitive effects of 5-HT_4_ agonists. Even if it represents an interesting target in neurological diseases, additional data are needed to precisely understand the multiple and subtle influences of this receptor in the hippocampus.

The 5-HT_2C_ receptor remains an important target in conditions associated with central dopaminergic transmission disturbances, but the multiplicity of brain regions engaged in this interaction is still a matter of discovery. Notably, Bombardi et al. focused on nicotine addiction and lateral habenula to show the increased activity of lateral habenula neurons with the 5-HT_2C_ receptor agonist Ro 60-0175, in acute nicotine-treated rats, was lost in chronic nicotine-treated rats [8]. This evidence adds to the findings on the 5-HT_2A_ receptor modulation of the nicotine effect on the lateral habenula published previously by the same authors [9,10]. Chronic nicotine seems to produce region-specific changes in 5-HT_2C_ receptor expression, such as in the prefrontal cortex. Lagière et al. illustrated the participation of 5-HT_2C_ receptors and the subthalamic nucleus and, more precisely, the hyperdirect pathway of the basal ganglia, linking the cortex to the substantia nigra via the subthalamic nucleus, in the oral responses induced by the dopamine D2/3 receptor agonist quinpirole in rats [11]. Indeed, quinpirole enhanced bouts of oral movements, and the electrophysiological response of substantia nigra neurons to cingulate cortex stimulation corresponds to the early excitatory response. As such, 5-HT_2C_ receptor antagonists reduced these effects and unmasked an increase in c-Fos expression in the subthalamic nucleus. The theatre of the 5-HT_2C_ receptor/dopamine transmission is much more widespread than initially thought [12].

In line with the identification of underrated brain networks participating in aberrant functions, Sere et al. addressed the role of the lateral hypothalamus in the absence seizures in the Genetic Absence Epilepsy Rats from Strasbourg (GAERS) [13]. In in vivo and in vitro experiments, using electrophysiological recordings and optogenetic tools, they found that spike-wave discharges (SWDs) spread in the lateral hypothalamus of GAERS while neurons of the lateral hypothalamus directly contact serotonergic neurons in the dorsal raphe nucleus. Through these experiments, the authors nicely recall the involvement of 5-HT neurons in absence seizures and, more broadly speaking, the possibility to find new serotonergic-based strategies for the treatment of epilepsy.

The 5-HT_2B_ receptor is a potential target in the CNS and for lung diseases. Löfdahl et al. summarized evidence pointing toward converging pathways in the fibrotic development in pulmonary fibrosis-interstitial lung diseases [14]. They reported interesting data, showing that 5-HT is involved in fibrosis, likely via the 5-HT_2B_ receptor, at least via non-canonical intracellular signaling pathways. The use of 5-HT_2B_ antagonists could have benefitted in the treatment of numerous interstitial lung diseases.

Other contributions witness the need to address, in depth, the 5-HT systems. Irritable bowel syndrome has been associated with alteration of the 5-HT system in the gut and also involves afferent neurons from the raphe nuclei. Mishima and Ishihara summarize data concerning 5-HT and irritable bowel syndrome, with special inference on the interaction of the microbiota and 5-HT function [15]. The hyperthermia that could be associated with a serotonergic syndrome or the use of some drugs can be reduced by the non-selective, serotonergic drug cyproheptadine. Petroianu covers the complex control exerted by 5-HT receptors in the control of thermoregulation and discusses the need to go beyond cyproheptadine to achieve a better response [16].

The last contribution concerns the reactivity of monoaminergic systems, including serotonin, to chemical agents used in agriculture. Bharatiya et al. reported the effect of chronic gavage of the pesticide fipronil in rats on the neurochemical level of monoamines in 30 brain regions [17]. The content of dopamine and 5-HT was dramatically reduced in the basal ganglia but enhanced in some cortical regions by fipronil. It was also associated with cortico-subcortical changes in connections between monoaminergic indexes.

We learned from this Special Issue that 5-HT_6_ receptor inverse agonists would be required to dampen the constitutive activity of 5-HT_6_ receptors that is promoting neurofibromatosis, while 5-HT_2B_ antagonists/inverse agonists would be useful in the treatment of interstitial lung diseases. Beyond these pragmatic results, the study of the 5-HT systems is still an incredible source of understanding of the pathophysiology of numerous diseases. It will imply a refinement in the existing neuropharmacological approaches to numerous brain diseases (e.g., use of 5-HT_1A_ and/or 5-HT_2A/2C_ compounds) and result in new conceptions of treatments via the 5-HT systems. We are confident that together with the authors of the Special Issue to have contributed to progress on the pathophysiology of the 5-HT systems.

## Data Availability

Not applicable.
